# Cortical responses to letters and ambiguous speech vary with reading skills in dyslexic and typically reading children

**DOI:** 10.1016/j.nicl.2021.102588

**Published:** 2021-02-12

**Authors:** Linda Romanovska, Roef Janssen, Milene Bonte

**Affiliations:** Maastricht Brain Imaging Center, Department Cognitive Neuroscience, Faculty of Psychology and Neuroscience, Maastricht University, Maastricht, Netherlands

**Keywords:** Dyslexia, Text-based recalibration, Audio-visual integration, Speech perception, Reading development, fMRI

## Abstract

One of the proposed issues underlying reading difficulties in dyslexia is insufficiently automatized letter-speech sound associations. In the current fMRI experiment, we employ text-based recalibration to investigate letter-speech sound mappings in 8–10 year-old children with and without dyslexia. Here an ambiguous speech sound /a?a/ midway between /aba/ and /ada/ is combined with disambiguating “aba” or “ada” text causing a perceptual shift of the ambiguous /a?a/ sound towards the text (recalibration). This perceptual shift has been found to be reduced in adults but not in children with dyslexia compared to typical readers. Our fMRI results show significantly reduced activation in the left fusiform in dyslexic compared to typical readers, despite comparable behavioural performance. Furthermore, enhanced audio-visual activation within this region was linked to better reading and phonological skills. In contrast, higher activation in bilateral superior temporal cortex was associated with lower letter-speech sound identification fluency. These findings reflect individual differences during the early stages of reading development with reduced recruitment of the left fusiform in dyslexic readers together with an increased involvement of the superior temporal cortex in children with less automatized letter-speech sound associations.

## Highlights

• Text recalibrates ambiguous speech perception in children with and without dyslexia.
• Dyslexia and poorer reading skills are linked to reduced left fusiform activation.
• Poorer letter-speech sound matching is linked to higher superior temporal activation.

## 1 Introduction

Reading is a fundamental skill in the modern day society. Once acquired, reading is an automated process facilitating employability, communication with others and ultimately technological and societal advances. We learn to read early on in our lives by mapping speech onto strings of symbols (text) and learning their meanings and associations. While this process goes smoothly for the majority of children, 5–10% of children are diagnosed with developmental dyslexia, a reading impairment characterised by difficulties in reading fluency and spelling despite adequate schooling opportunities, motivation, intelligence and sensory abilities (Lyon et al., 2003, Peterson and Pennington, 2012).

Dyslexia is a specific learning disorder that is heritable, has a neurobiological basis and is heterogeneous in its cognitive-behavioural manifestation (Astrom et al., 2007, Pennington, 2006, Schumacher et al., 2007, Snowling and Melby-Lervåg, 2016, Van Bergen et al., 2012, van Bergen et al., 2014). Despite this variability, a proposed core deficit is impaired manipulation of speech sounds - i.e. phonological skills (Goswami, 2003, Lyon et al., 2003, Shaywitz et al., 1998, Snowling, 1980, Snowling, 2013). It has been suggested that children and adults with dyslexia have less intact phonological representations and/or have difficulty accessing these representations (Bonte et al., 2007, Noordenbos and Serniclaes, 2015, Ramus and Szenkovits, 2008). An extension of this view proposes that dyslexia involves impaired (e.g. less automatized) mapping of letters and speech sounds (Aravena, 2017, Blomert, 2011, Blomert and Willems, 2010, Kronschnabel et al., 2014; however see Clayton and Hulme, 2017, Nash et al., 2016), with robust letter-speech sound associations constituting a pillar of successful reading.

As a child learns to read, the left occipito-temporal cortex becomes increasingly specialised for text (Ben-Shachar et al., 2011, Brem et al., 2009, Dehaene-Lambertz et al., 2018, Maurer et al., 2006) and linked to speech processing areas, including the superior temporal cortex (STC; Dehaene et al., 2015, Schlaggar and McCandliss, 2007). This link is illustrated by a cross-modal enhancement of STC activation by the combined presentation of letters and speech sounds (Van Atteveldt and Ansari, 2014, Van Atteveldt et al., 2004), particularly in relatively transparent orthographies such as Dutch. More specifically, functional magnetic resonance imaging (fMRI) studies have shown that presenting typical readers with matching (congruent) compared to non-matching (incongruent) letter-speech sound pairs elicits increased activation in STC (Blau et al., 2010, Karipidis et al., 2017, Van Atteveldt and Ansari, 2014). Employing this paradigm in dyslexic readers has revealed reduced letter-speech sound congruency effects in the left STC in at risk pre-readers (Plewko et al., 2018), and in children (Blau et al., 2010), adolescents (Kronschnabel et al., 2014) and adults (Blau et al., 2009, Ye et al., 2017) with dyslexia compared to their age-matched typically reading peers. Similarly, reduced activation has also been reported in higher-order visual areas in studies employing rhyme judgment tasks investigating reading-skill dependent cross modal (McNorgan and Booth, 2015) and unimodal (Hoeft et al., 2007) processing of rhyming versus non-rhyming word pairs. Together, these studies point to aberrant neural processing of letters and speech sounds in readers with dyslexia compared to typical readers.

Congruency manipulations inherently rely on culturally learnt letter and speech sound associations. However, in children and adults with dyslexia, these links may be represented or automatized differently than in typical readers, potentially confounding the observed results. An alternative way to investigate audio-visual integration of text and speech sounds can be found in text-based recalibration. In this task, ambiguous speech is combined with disambiguating text to explore audio-visual integration of the two modalities. The task consists of two distinct parts – audio-visual exposure blocks followed by auditory-only post-test trials. During the exposure blocks, an ambiguous speech sound /a?a/ midway between /aba/ and /ada/ is combined with disambiguating “aba” or “ada” text. The visual stimuli serve as a “touchstone” for the perceptual system and aid the audio-visual integration of ambiguous speech and visual input. If the two modalities are successfully combined, the perception of the ambiguous sound will temporarily be biased towards the visual stimulus. The extent of the perceptual bias is tested in subsequent post-test trials. Here, participants are presented with the ambiguous sound in isolation (i.e. no visual input) and asked to respond if they perceive the sound as /aba/ or /ada/. Repeated exposure to the ambiguous speech sound /a?a/ in combination with e.g. disambiguating “aba” text, shifts the perception of the speech sound towards /aba/ as illustrated by a larger proportion of /aba/ responses in the post-test trials. Similarly, combining the ambiguous /a?a/ sound with “ada” text biases the later perception of the same speech sound towards /ada/ (Keetels et al., 2018, Keetels et al., 2016). The perceptual bias represents a shift in the participant’s phoneme boundary towards the visual modality and is referred to as recalibration. Recalibration is described as a perceptual effect that relies on short-term audio-visual learning mechanisms (Samuel and Kraljic, 2009, Vroomen and Baart, 2012) and temporarily maps the ambiguous sound onto a pre-defined phoneme category (e.g. /a?a/ mapped onto /aba/). A number of visual stimuli have been shown to elicit recalibration including lip-read speech (Bertelson et al., 2003, Ullas et al., 2020a, Vroomen and Baart, 2012), spoken word context (Norris et al., 2003, Ullas et al., 2020a), overt speech articulation (Scott, 2016), and most recently, text (Bonte et al., 2017, Keetels et al., 2016).

The use of text to disambiguate speech is of particular interest for dyslexia research, as this allows exploring audio-visual associations between letters and speech sounds while sidestepping task or stimulus factors involving explicit matching between specific speech sounds and text. In a study employing text-based recalibration, adults with and without dyslexia were exposed to ambiguous speech /a?a/ in combination with either a disambiguating video of a speaker articulating ‘aba’ or ‘ada’, or using “aba” or “ada” text. Intriguingly, while typical readers showed significant recalibration effects following both video and text, readers with dyslexia only showed significant recalibration when videos were used as the disambiguating visual stimuli (Keetels et al., 2018). These findings point to a specific letter-speech sound integration deficit in dyslexia rather than a general deficit in audio-visual integration. However, recent findings in 8–10 year old children employing the same paradigm, surprisingly showed comparable text-induced recalibration in typical and dyslexic readers (Romanovska et al., 2019). It has been proposed that children are particularly sensitive to text within the first few years of reading instruction (Fraga González, 2015, Froyen et al., 2008, Maurer et al., 2008, Price and Devlin, 2011, Žarić et al., 2014). Because the proposed ‘peak’ text sensitivity period falls within the age range of the children tested in the abovementioned study, the observed discrepancy in findings between children and adults with dyslexia may point to a developmental aspect of text-based recalibration. Indeed, previous research employing lip-read speech as the disambiguating visual stimulus has demonstrated a robust effect in 8- but not 5-year-olds (van Linden and Vroomen, 2008). The authors attributed this to less proficient lip-reading in the 5-year-olds and suggested that increased experience with lip reading (and by extension speech processing) likely has an effect on recalibration. In addition to possible effects of a history of reading problems, developmental differences in letter-speech sound processing may similarly underlie the reported differences in text-based recalibration between adults and children with dyslexia (Romanovska et al., 2019).

In the current fMRI study, we aimed to explore the neural mechanisms underlying audio-visual integration of ambiguous speech and text using text-based recalibration in 8–10 year-old children with and without developmental dyslexia. We were particularly interested in investigating group differences in cortical activation, given the comparable task performance behaviourally. We focused our analysis on the audio-visual exposure blocks, where previous fMRI recalibration studies in adults using lip-read (Kilian-Hütten et al., 2011a) and text (Bonte et al., 2017) stimuli have shown the involvement of a network of brain areas related to audio-visual processing of speech and text. The behavioural responses provided in the post-test trials were assessed to investigate the recalibration effect in both groups of children while they performed the task in the MRI scanner. In line with behavioural findings (Romanovska et al., 2019), we did not expect to see any difference in the recalibration effect between children with and without dyslexia. We did, however, expect differences in brain activation between the groups, with dyslexic readers showing less cortical activation in reading-related auditory and visual regions compared to their typically reading peers. We first explored the cortical activation pattern during the exposure blocks in a whole-brain analysis. We then furthered these analyses by focusing on regions of interest (ROIs) typically associated with audio-visual integration and reading based on children’s brain activity during an adapted version of the congruency manipulation paradigms (e.g. Blau et al., 2010, Plewko et al., 2018), a passive viewing/listening task. Investigating cortical activation in these regions with a novel audio-visual integration task allowed to explore the hypothesis of letter-speech sound integration difficulties in dyslexic readers during short-term perceptual mapping of ambiguous speech to text. Finally, we performed correlation analyses to explore the links between cortical activation within the ROIs and children’s reading and phonological skills.

## 2 Methods

### 2.1 Participants

Twenty-nine children with dyslexia (mean age 9.4 ± 0.6 years; 15 females) were recruited from a specialized institute for dyslexia healthcare, and forty-three typically reading children (mean age 8.9 ± 0.7 years; 24 females) were recruited from local elementary schools. Data of five dyslexic children were excluded from the analyses due to excessive head motion during the fMRI measurement resulting in poor data quality. The remaining 23 children with dyslexia (2 left-handed) were matched with 23 typical readers (1 left-handed) for age, gender and scores on a non-verbal subtest (block design) of the Dutch version of the Wechsler Intelligence Scale for Children-III (WISC-III-NL; Kort et al., 2005). Twenty of the children (8 dyslexic readers) had taken part in the behavioural text-based recalibration experiment (Romanovska et al., 2019) and were subsequently invited to participate in the fMRI study. The remaining twenty-six children (14 dyslexic readers) were recruited after the behavioural study was completed. Because we were interested in exploring the text-based recalibration effect in the MRI scanner and behaviourally (offline, on a laptop computer as in the behavioural study), these twenty-six children completed the offline text-based recalibration task after the scanning session (total duration 10 min).

All children were native Dutch speakers with no reported hearing impairments, normal or corrected to normal vision, and no history of diagnosed comorbid developmental or neurological disorders. The dyslexia diagnosis was given by the specialised dyslexia institute based on the results of an extensive cognitive psycho-diagnostic testing procedure and all scored at or below the 10th percentile on standardized reading measures. The dyslexic readers were within the first three months of dyslexia treatment. Parents provided written informed consent for participation in the study in accordance with the declaration of Helsinki. Children received a present and a picture of them in the mock scanner as participation reward. The experiment was approved by the ethics committee of the Faculty of Psychology and Neuroscience, Maastricht University.

#### 2.1.1 Literacy and cognitive skills

All participants completed computerized reading, letter-speech sound identification, and phoneme deletion tasks of the 3DM test battery (Dyslexia Differential Diagnosis; Blomert and Vaessen, 2009), as well as two sub-tests of the WISC-III-NL – verbal (similarities) and non-verbal (block design). The reading task was sub-divided into three parts – reading of high frequency, low frequency and pseudo words. Reading fluency was calculated as the total number of words read within 90 s (30 s per category). During the letter-speech sound identification task, the children were presented with a phoneme aurally via headphones and asked to indicate the corresponding letter(s) out of 4 possibilities on the computer screen, via button press. During the phoneme deletion task, the participants were presented with a pseudo word via headphones, followed by a phoneme from this pseudo word and asked to say out loud what the pseudo word would sound like without the phoneme (e.g. say /dauk/ without the /d/). All task instructions were simultaneously presented on the computer screen and aurally via headphones, instructing the children to perform the tasks as quickly and accurately as possible. For letter-speech sound identification and phoneme deletion, fluency scores constitute the number of correctly completed items in each task out of the maximum number of items (90 for letter-speech sound identification, 28 for phoneme deletion).

Group characteristics and comparisons between children with and without dyslexia using one-way ANOVA are shown in Table 1. As expected, the children with dyslexia scored significantly lower on the reading and phonological tasks compared to typical readers. The groups differed in the non-verbal IQ sub-test, with dyslexic children having slightly lower scores on average. Importantly however, all children were within, or indeed somewhat above the norm on this measure.

**Table 1:** Descriptive statistics of the sample and group comparisons of dyslexic and typical readers.

Group	Dyslexic readers			Typical readers			Dyslexic vs. Typical	
Age (SD) Gender ratio(m/f)	9.4 (0.6) 10:13			9.1 (0.7) 10:13			readers	
Reading fluency scores^1^	*M*	*SD*	*Range*	*M*	*SD*	*Range*	*F(1,45)*	*p*
Word reading	77.82	20.13	40–111	121.17	27.97	74–178	36.39	**0.000**
Word reading [T]2	34.52	6.20	22–46	53.82	11.87	30–80	47.75	**0.000**
Letter-speech sound identification	42	3.41	33–45	42.95	1.55	40–45	1.49	0.227
Letter-speech sound identification [T]	44	9.73	24–59	54.43	5.31	43–64	20.38	**0.000**
Phoneme deletion	13.30	6.20	5–23	17.34	4.05	8–23	8.17	**0.006**
Phoneme deletion [T]	36	13.90	0–56	53.21	11.75	30–74	20.56	**0.000**
IQ norm scores^3^								
Verbal (similarities)	11.56	2.25	8–16	15.04	1.66	12–18	35.46	**0.000**
Non-verbal (block design)	10.21	2.66	7–17	11.82	1.99	7–16	5.38	**0.025**
Age (months)	115.17	9.18	99–132	110	9.18	96–126	3.65	0.063

^1^Raw scores, number of correct items across three sub-groups (high-frequency, low-frequency and pseudo words) per 90 s, number of correct responses out of 90 items (letter-speech sound identification) or 28 items (phoneme deletion).

^2^t-Scores, age-appropriate norm scores mean 50, SD = 10.

^3^Age-appropriate norm scores, mean = 10, SD = 3.

### 2.2 Stimuli

The speech stimuli for the recalibration task consisted of recordings of a native male Dutch speaker pronouncing the speech sounds /aba/ and /ada/ (see Bertelson et al., 2003 for a detailed description). Both speech sounds lasted 650 ms and were used to create a nine-token continuum ranging from a clear /aba/ sound to a clear /ada/ sound by changing the second formant (F2) in eight steps of 39 Mel using PRAAT software (Boersma and Weenink, 2001). The visual stimuli consisted of the written counter-parts of the speech sounds, namely “aba” and “ada” text presented in white at the center of a black screen in ‘Times New Roman’ font (font size 50). The auditory and visual stimuli were presented using Presentation software (Version 18.1, Neurobehavioral Systems, Inc., Berkeley, CA, United States).

In addition to the fMRI recalibration experiment, the children performed a passive viewing/listening task with unimodal and bimodal presentation of letters and speech sounds (adapted from Blau et al., 2010). The task included four stimulus conditions: audio-visual congruent (matching letters and speech sounds), audio-visual neutral (meaningless symbols and speech sounds), auditory-only and visual-only. Speech stimuli for this task consisted of 10 Dutch consonant–vowel syllables produced by two female native Dutch speakers (/ba/, /bi/, /bu/, /da/, /fi/, /fu/, /si/, /su/, /ti/, /tu/; a subset from Correia et al., 2015) and 3 Dutch vowels produced by two native Dutch children (one boy, one girl; /a/, /i/, /u/; a subset from Bonte et al., 2014). The stimuli were recorded in a soundproof chamber and post-processed using PRAAT software (Boersma and Weenink, 2001). All stimuli were digitized at a sampling rate of 44.1 kHz (16 bit resolution), bandpass filtered (80 – 10.5 kHz) and down sampled to 22.05 kHz. Stimulus length was equalized to 350 ms for the vowels and 340 ms for the consonant–vowel syllables using PSOLA (75 – 400 Hz for the F0 contour). Sound intensity level was equalized across stimuli and adjusted to the in-scanner headphone system (Sensimetrics, model S14, www.sens.com).

The visual stimuli for the congruent and visual-only condition were visual letters/syllables corresponding to the speech sounds, presented in white at the centre of a black screen in ‘Verdana’ font (font size 50). The visual stimuli for the neutral condition consisted of 15 meaningless symbol combinations containing two or three elements presented in a pseudo-randomized order ensuring that no speech sound-symbol associations could be made. The symbols were presented in white on a black screen and their size was matched to the text stimuli to ensure comparable stimulus properties. In the visual-only blocks, the letters/syllables were presented in isolation, whereas in the auditory-only blocks only the speech sounds were presented while the participants fixated on a white fixation cross in the centre of a black screen. An orthogonal task was employed to assure attention and included catch trials matching the four conditions (similarly to Blau et al., 2010). The catch trials consisted of a cartoon monster (visual stimulus) and a recording of a female native Dutch speaker saying /Hello!/ (auditory stimulus) presented in isolation in the visual- and auditory-only blocks respectively. A combination of both modalities was presented in the congruent and neutral blocks.

### 2.3 Experimental design and procedure

Prior to the MRI experiment, all children were trained in a mock scanner to get acquainted with the scanning environment, practice the recalibration task and help reduce head motion during data acquisition. Upon arrival, we explained the tasks that the children would be performing in the MRI scanner, namely the recalibration and passive viewing/listening task. The children then practiced in the mock scanner to get acquainted with the use of the MR compatible headphones (Sensimetrics, model S14, https:www.sens.com) and button boxes. During the practice, all children completed a pre-test (see 2.3.1) followed by one run of the recalibration task consisting of one “aba” and one “ada” exposure block, each followed by four post-test sounds. The children then completed motion training in order to improve subsequent (f)MRI data quality. This consisted of placing a headband containing a motion sensor on the forehead of each child while they watched a cartoon inside the mock scanner. The sensor was calibrated to tolerate 2 degrees of motion along the horizontal and vertical planes, as soon as this threshold was exceeded, the cartoon paused and shrank until the child was lying still again. This helped illustrate how still the children should aim to lie during the MRI experiment. The duration of the mock training session was approximately 20 min. The children then completed a 1 h 15 min MRI experiment and 45 min behavioural testing after the scanning session in which they completed the reading tasks and two subsets of the WISC-III-NL. While the allotted scanning time was 1 h and 15 min, the data acquisition only took 45 min in total. The rest of the time was used for short breaks in between tasks and taken up by placing the participants in the scanner and taking them out of the scanner. Total testing time amounted to 2 h and 45 min including two breaks – a 10 min break after the mock scanner training and a 15 min break after the MRI experiment.

#### 2.3.1 Pre-test

**Fig. 1:**
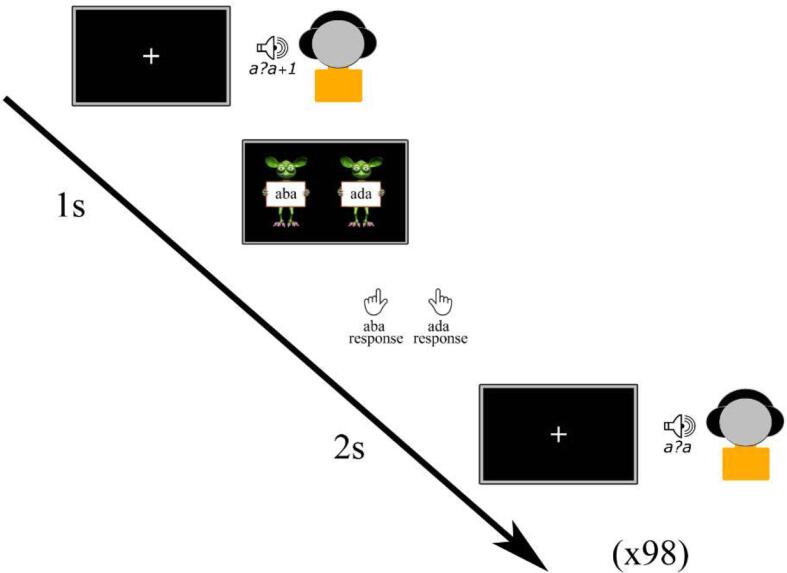
Pre-test.

During the training session, each child completed a pre-test to determine the individual most ambiguous sound for subsequent use in the recalibration task. The children were presented with all 9 sound tokens along the /aba/-/ada/ continuum a total of 98 times in a randomized order, with the 7 ambiguous sounds presented more frequently than the clear /aba/ and /ada/ sounds (see e.g. Bertelson et al., 2003, Kilian-Hütten et al., 2011b, Vroomen et al., 2004). The participants were instructed to pay close attention to each sound and indicate whether they perceived that sound as /aba/ or as /ada/, by pressing the left or right innermost button of a button box with their left/right index finger following a response cue (Fig. 1). The response cues consisted of text “aba” (left) and “ada” (right), held up by cartoon monsters created using the Monster Workshop content pack of the iClone 6 software (https://www.reallusion.com/). During the presentation of the speech sounds, the children viewed a black screen with a white fixation cross followed by the response cue 1 s later. Each trial was terminated after the child provided a response, triggering the presentation of the subsequent speech sound after 2 s. The total duration of the pre-test was approximately 5 min.

The most ambiguous speech sound was determined based on the proportion of /aba/ responses to each token along the /aba/-/ada/ continuum and was identified as the sound with an /aba/ versus /ada/ response proportion closest to 0.5 representing the phoneme boundary (Romanovska et al., 2019, Vroomen et al., 2004). This individually determined most ambiguous sound was subsequently used in the audio-visual exposure blocks and post-test trials of the recalibration task. In the post-trials, next to the most ambiguous sound, we also presented its flanking sounds /a?a/+1 and /a?a/−1 along the /aba/-/ada/ continuum.

#### 2.3.2 Recalibration task

**Fig. 2:**
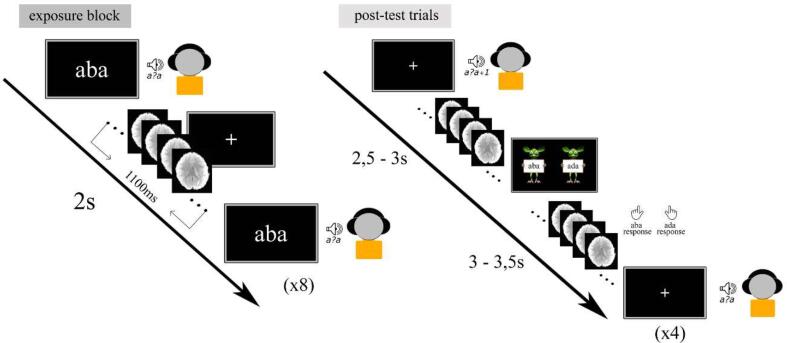
Text-based recalibration paradigm in the MRI environment. Left panel: timings of audio-visual stimulus presentation (8 stimuli per block) during the exposure blocks, with a 2 s inter-stimulus interval (TR) and a 1100 ms acquisition period (TA), leaving 900 ms silent gaps for stimulus presentation. Right panel: timings of the subsequent post-test trials (4 stimuli per block), with jittered periods before and after the response cue presentation and the time-window in which the participants provided their response. Also here a TR of 2 s, and a TA of 1100 ms was used leaving 900 ms silent gaps for post-test sound presentation.

The recalibration paradigm consisted of audio-visual exposure blocks and subsequent post-test trials (Fig. 2). During each exposure block, the children were presented with text “aba” or “ada” in combination with the individually determined most ambiguous speech sound /a?a/ for a total of 8 times. The “aba” and “ada” exposure blocks were presented in a pseudo-randomised order, ensuring that each type of exposure block was repeated no more than twice in a row. The audio-visual stimuli were presented simultaneously (relative SOA of 0 ms), the duration of the auditory stimuli was 650 ms and visual text was presented for 1 s. The inter-trial interval between subsequent audio-visual exposure trials was set to 2 s (1 TR). During the audio-visual exposure blocks, children were instructed to pay close attention to the speech sounds and text without providing a response.

Each exposure block was followed by four auditory-only post-test trials the onset of which was jittered to be an average of 10 s (4–6 TR). The jittered period between exposure blocks and post-test sounds served as the baseline in subsequent statistical comparisons and consisted of a white fixation cross in the middle of a black screen. The post-test trials were presented in a randomized order with the most ambiguous sound /a?a/ presented twice, and each of the flanking sounds /a?a/+1 and /a?a/-1 on the /aba/-/ada/ continuum presented once. Each post-test trial was followed by a response cue containing cartoon monsters (Fig. 2). The onset of the response cue was jittered 2,5–3 s with respect to the post-test sound and lasted 3 s. The subsequent post-test trial was presented 3–3,5 s following the response cue. The total ITI between post-test trials was 6 s (3 TR). Children were instructed to listen carefully to the post-test sound and respond whether they perceived it as /aba/ or as /ada/ upon the presentation of the response cue using the MR compatible button boxes. The responses were made by pressing the innermost button of the button box with the left/right index finger, as practiced in the mock scanner. Children completed a total of four runs of the recalibration task, corresponding to 24 audio-visual exposure blocks (12 with “aba” text and 12 with “ada” text) and 4*24 post-test trials. All auditory and audio-visual stimuli were presented during a 900 ms silent gap in volume acquisitions.

#### 2.3.3 Passive viewing/listening task

At the end of the fMRI session, the children completed a single run of a passive viewing/listening task with four stimuli blocks presented in a pseudo-randomised order: bimodal speech sounds and text (congruent), bimodal speech sounds and meaningless symbols (neutral), unimodal speech sounds, and unimodal text. Each block contained 6 stimuli presented once every 2 s (1 TR). Subsequent blocks were separated by a jittered rest period of 12 s on average (5–7 TR) which served as the baseline and consisted of a white fixation cross in the middle of a black screen. To ensure children were paying attention, an orthogonal task using pseudo-randomized cartoon monster catch trials was included (similar to Blau et al., 2010). There was a total of 6 catch trials matched for the modality of the stimuli blocks − 3 bimodal catch trials and 3 unimodal trials (2 visual). During the bimodal catch trials the children saw a cartoon monster and simultaneously heard the monster say /Hello!/, during the unimodal catch trials they only saw or heard the monster. The children were instructed to pay close attention to the stimuli because a cartoon monster was hiding somewhere between them and press the right innermost button of the button box with their right index finger as soon as they heard and/or saw the monster.

### 2.4 Statistical analyses behavioural data

The behavioural data were analysed using SPSS version 26.0 (IBM Corp., Armonk, NY, United States). In addition to the behavioural data collected while children were performing the recalibration task in the scanner, we also investigated each child’s performance on the recalibration task outside of the scanner during the behavioural experiment (i.e. offline data). We were thus able to compare recalibration effects in and out of the MRI scanner for each child. For both, in scanner and offline data, RM ANOVA analyses were performed investigating group effects of dyslexia diagnosis on the performance of the recalibration task. The ANOVA models included the type of exposure block (“aba” vs “ada”) and type of post-test sound (/a?a/,/a?a/+1,/a?a/-1) as within subject factors and dyslexia (dyslexic vs typical readers) as the between subjects factor. For the offline data, an additional between subjects factor for task order was included (before vs after MRI) to test for potential differences in task performance between children who completed the behavioural text-based recalibration task before the scanning session compared to the children who completed the task afterwards. The Greenhouse-Geisser correction of the degrees of freedom was used for conditions violating the sphericity assumption.

To investigate the association between children’s letter-speech sound processing, phonological, and reading skills, and cortical activation during the audio-visual exposure blocks we performed correlation analyses. Behavioural measures included children’s non-standardized raw scores of word reading fluency, letter-speech sound identification fluency and phoneme deletion fluency. Prior to running the analyses, all data were assessed for outliers using boxplots in SPSS. The analyses identified two dyslexic readers as outliers in the letter-speech sound fluency task (lower quartile plus 1.5 times inter-quartile range). These participants were excluded from the correlation analyses exploring the association between cortical activation and letter-speech sound fluency. All other correlations were performed on the full sample of 46 participants. Bivariate Pearson correlations were computed one at a time (i.e. for each behavioural measure separately) using the built-in ANCOVA analyses module in BrainVoyager 20.6 based on the average individual t-statistics extracted per participant from a pre-defined region of interest. The correlations were corrected for multiple comparisons by applying the False Discovery Rate (FDR) correction using MATLAB.

### 2.5 MRI measurements

Brain Imaging was performed with a Siemens Prisma 3 T MRI scanner (Siemens Medical Systems, Erlangen, Germany) using a 64-channel head–neck coil. Five functional runs were acquired (2,5 mm × 2,5 mm × 2,5 mm resolution) with a multi-band factor of 5 echoplanar-imaging (EPI) sequence (repetition time [TR] = 2000 ms, acquisition time [TA] = 1100 ms, field of view [FOV] = 210 mm × 210 mm, echo time [TE] = 35.8 ms). Each volume consisted of 50 slices (no gap), covering the whole brain. The recalibration task was made up of four 5 min runs and the passive viewing/listening task consisted of one 7 min functional run. The speech stimuli were presented binaurally at a comfortable listening level via MR compatible headphones (Sensimetrics, model S14, www.sens.com), in the 900-ms silent gap between consecutive volume acquisitions. Additionally, a high-resolution structural scan (1 mm × 1 mm × 1 mm) using a T1-weighted three-dimensional MPRAGE sequence ([TR] = 2300 ms, [TE] = 2.98 ms, 192 sagittal slices) was acquired.

#### 2.5.1 fMRI pre-processing

Data pre-processing and analyses were performed using BrainVoyager QX version 2.8, BrainVoyager 20.6 and 21.4 (Brain Innovation, Maastricht, The Netherlands) and custom MATLAB routines (The MathWorks, Inc., Natick, MA, United States). The functional data underwent 3D motion correction with respect to the first volume of the first functional run (trilinear sinc interpolation), slice scan time correction and high pass temporal filtering (5 cycles per time course recalibration runs / 7 cycles passive viewing/listening paradigm). The anatomical data underwent manual inhomogeneity correction to improve white matter-grey matter boundary segmentation and was transformed into Talairach space (Talairach and Tournoux, 1988). The functional data were co-registered to the anatomical data, transformed into Talairach space, re-sampled to 3 mm *iso*-voxel resolution and spatially smoothed using a 6 mm FWHM Gaussian kernel. Volumes of functional runs affected by excessive head motion (≥3 mm translation/rotation in any direction) were removed from the run, if the number of affected volumes exceeded 20%, the run was excluded from further analyses. A one-way ANOVA of the average motion statistics for each of the 3 translation and rotation parameters did not reveal significant differences in motion between children with and without dyslexia (all F ≤ 1.85).

For each child, individual cortical surface representations were automatically constructed based on the white matter-grey matter boundary, manually adjusted, and aligned using cortex based alignment employing a moving-target group average based on curvature information resulting in an anatomically-aligned group-average 3D cortical representation (Frost and Goebel, 2012). Each participant’s functional data were projected onto their cortical surface creating surface-based time courses. All functional data were subsequently analysed per hemisphere at the surface level using the group-aligned average cortical surfaces.

### 2.6 Region of interest (ROI) definition

The regions of interest were defined based on cortical activation during the congruent vs baseline condition in the passive viewing/listening task. Three participants did not complete this task (2 dyslexic readers) and data of six participants were excluded due to excessive head motion (3 dyslexic readers). The individual maps of the remaining 37 participants were each thresholded at p < 0.05 (uncorr.; fixed cluster threshold of 25 mm^2^), anatomically aligned and used to create group-based probabilistic maps (Frost and Goebel, 2012). The resulting group maps were thresholded at 60%, thus including regions of 60% subject overlap at a fixed group cluster threshold of 20 mm2 for each separate group (dyslexic and typical readers). We chose to perform these analyses for each group separately to delineate regions of interest that may or may not be specific to dyslexic or typical readers. The resulting group maps showed comparable regions of consistent activation in both groups, albeit with lower inter-subject consistency across dyslexic readers. Because of the involvement of comparable regions, we decided to create ROIs based on the combined probabilistic maps across groups. The choice for 60% overlap was based on setting a minimum criterion that included consistent activity in auditory and visual brain regions in more than half of the individual children. In practice, this threshold was especially driven by the relatively large inter-individual variability in the exact location of children’s activity in the ventral visual cortex. This variability is in line with the proposition that the recruitment of the ventral visual areas is still variable around age 9, since children have not yet made a switch to fully automatized text processing at this age (Ehri, 2005, Pugh et al., 2001). In fact, at the 60% overlap threshold the ventral visual region only occurred in the map of the typical readers, which may relate to the fact that typical readers on average were closer to approaching automatized reading. Combining the ROI maps of both groups yielded four ROIs typically associated with audio-visual integration and reading including the left fusiform gyrus/occipito-temporal sulcus, bilateral superior temporal gyri (STG) and right frontal cortex (Fig. 5). These regions were used in subsequent correlation analyses and group comparisons of cortical activation during the audio-visual exposure blocks in the recalibration task.

### 2.7 Whole brain univariate fMRI analysis

Cortical activation was assessed employing random effects (RFX) general linear model (GLM) analyses using the individual surface-based time courses of all participants. The model included one predictor for each type of exposure and post-test blocks (“aba”, “ada”; 4 predictors) as well as z-transformed motion predictors as variables of no interest to improve the signal to noise ratio. The number of runs included in the RFX analyses varied by participant due to excessive head motion (6 participants, 5 dyslexic readers) or technical difficulties during data acquisition (1 typical reader). The total number of recalibration task runs was 175 (86 runs dyslexic readers, 89 runs typical readers). Subsequent functional contrast maps (*t*-statistics) were calculated based on predictors for both exposure blocks taken together (“aba” and “ada”) compared to the fixation cross baseline. These maps were corrected for multiple comparisons using an FDR threshold of q < 0.05 and contrasted in whole brain group comparisons of dyslexic versus typical readers.

### 2.8 ROI analysis

In addition to group comparisons at the whole-brain level, we explored cortical activation during the audio-visual exposure blocks in four ROIs: bilateral STG, left fusiform and right frontal cortex in children with and without dyslexia. This was achieved by running ROI ANOVA analyses in BrainVoyager 20.6 comparing the t-statistic values within each ROI between children with and without dyslexia. We additionally conducted ANCOVA analyses in each ROI to check for potential confounding effects of individual differences in age and in scores on verbal and non-verbal sub-tests of the WISC-III-NL, as these showed significant differences between the groups (WISC sub-tests) or approached statistical significance (age). In order to explore potential links between reading skills and cortical activation within the ROIs, we also performed correlations of the individual t-statistics and reading skills.

## 3 Results

### 3.1 Behavioural results offline experiment

**Fig. 3:**
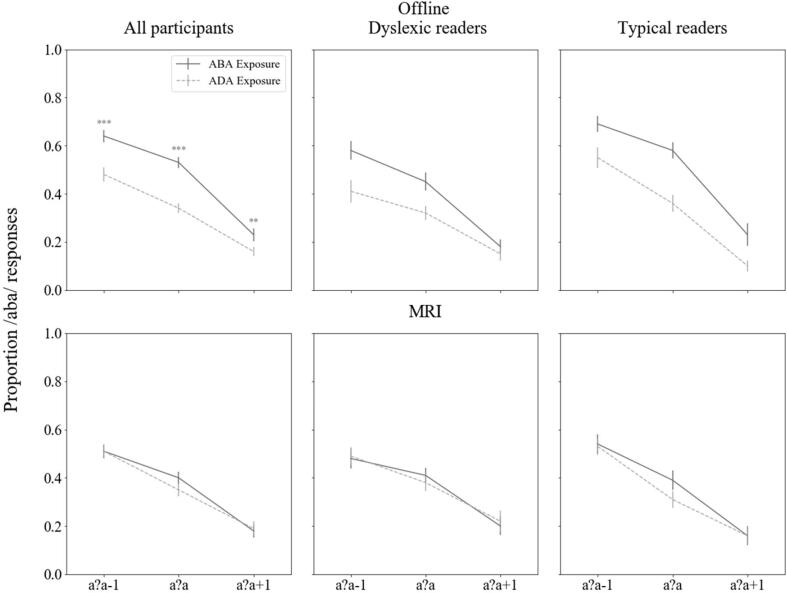
Behavioural text-based recalibration results; Top panel: outside the MRI scanner; bottom panel: in the MRI scanner; The graphs show /aba/ response proportions for the /a?a/-1, /a?a/ and /a?a/+1 post-test sounds following an “aba” versus “ada” exposure block. Vertical bars = standard error; **p ≤ 0.01 ***p ≤ 0.001.

Visual inspection of the offline data revealed a clear recalibration effect across all participants as well as within the matched groups of typical and dyslexic readers (Fig. 3 top panel). The children were more likely to perceive the ambiguous post-test sounds as /aba/ following an “aba” exposure block (dark grey line Fig. 3 top panel) and as /ada/ following an “ada” exposure block (light grey dashed line Fig. 3 top panel). The effect was especially pronounced for the most ambiguous speech sound /a?a/: proportion of /aba/ versus /ada/ responses 0.51 vs 0.33 across participants, 0.45 vs 0.32 in dyslexic readers, 0.57 vs 0.35 in typical readers.

The recalibration effect across groups was confirmed by a 2 (exposure) × 3 (post-test sounds) RM ANOVA with between subject factors dyslexia and task order. Two participants (1 dyslexic reader) did not complete the offline behavioural experiment, thus this analysis included data of 44 out of the 46 participants. Results showed significant main effects of exposure [F(1,40) = 27.88, p < 0.001] and post-test sound [F(1,64) = 146.73, p < 0.001, Greenhouse-Geisser corrected], as well as a significant exposure × post-test sound interaction [F(2,80) = 5.99, p = 0.004], showing that children’s /aba/ response proportions differed depending on the type of exposure block (“aba” versus “ada”) and post-test sound (/a?a/, /a?a/+1 versus /a?a/-1). Post hoc comparisons of /aba/ response proportions following the two types of exposure blocks across all participants confirmed a significant difference for each of the post-test sounds following “aba” vs “ada” exposure, reflecting a significant recalibration effect across children [/a?a/: M = 0.51, SD = 0.17, M = 0.33, SD = 0.15, *t*(43) = -5.33, *p* < 0.001; /a?a/+1: M = 0.20, SD = 0.18, M = 0.12, SD = 0.12, *t*(43) = -2.68, *p* = 0.01; /a?a/-1: M = 0.64, SD = 0.18, M = 0.48, SD = 0.21, *t*(43) = -4.51, *p* < 0.001].

The analyses also revealed a main effect of dyslexia [F(1,40) = 4.64, p < 0.05], which could either reflect a group difference in the magnitude of the recalibration effect or in their overall /aba/ versus /ada/ response proportions. To test the first possibility, we conducted a one-way ANOVA analysis comparing the magnitude of the recalibration effect between readers with and without dyslexia. Results showed no significant difference in recalibration effects between groups [F(1,43) = 0.927, *p* = 0.341]. The main effect of dyslexia thus likely points to a difference in overall response proportions. Indeed, the average /aba/ versus /ada/ response proportions were somewhat lower in dyslexic (M = 0.33) compared to typical readers (M = 0.48), indicating that dyslexic readers were more likely to report perceiving the ambiguous post-test sounds as /ada/ than typical readers.

As for possible effects of performing the behavioural task prior to or after the MRI scan, the RM ANOVA showed no main effect of task order (F = 2.77), and no significant interactions with dyslexia or dyslexia and task order (F ≤ 3.29), indicating that neither dyslexia diagnosis, nor task order or their interaction had a significant effect on the recalibration results. The results did include a significant task order × post-test sound interaction [F(2,64) = 6.87, p = 0.002] suggesting that the /aba/ versus /ada/ response proportions to the different post-test sounds were differentially influenced by whether the participants performed the task before or after the MRI (see slopes for the “aba” and “ada” exposure blocks in supplementary Fig. S1). Given the lack of main- or other interaction effects with task order, these findings do not indicate differences in recalibration effects.

### 3.2 Behavioural results in the scanner

The behavioural results of the same participants in the MRI scanner showed a marked decrease in the magnitude of the recalibration effect (Fig. 3 bottom panel). The proportions of /aba/ to /ada/ responses to the most ambiguous sound /a?a/ were 0.39 vs 0.35 across participants, 0.41 vs 0.38 in dyslexic readers and 0.38 vs 0.31 in typical readers. A 2 (exposure) × 3 (post-test sounds) RM ANOVA across all subjects showed only a significant main effect of post-test sound [F(1,65) = 65.83, p < 0.001, Greenhouse-Geisser corrected], none of the other main or interaction effects were statistically significant (F ≤ 2.3). These results indicate that, while all participants responded differently to each post-test sound (downward slopes in Fig. 3 bottom panel), the recalibration effect was not significant in either the dyslexic or typical readers.

### 3.3 fMRI activity during audio-visual exposure

**Fig. 4:**
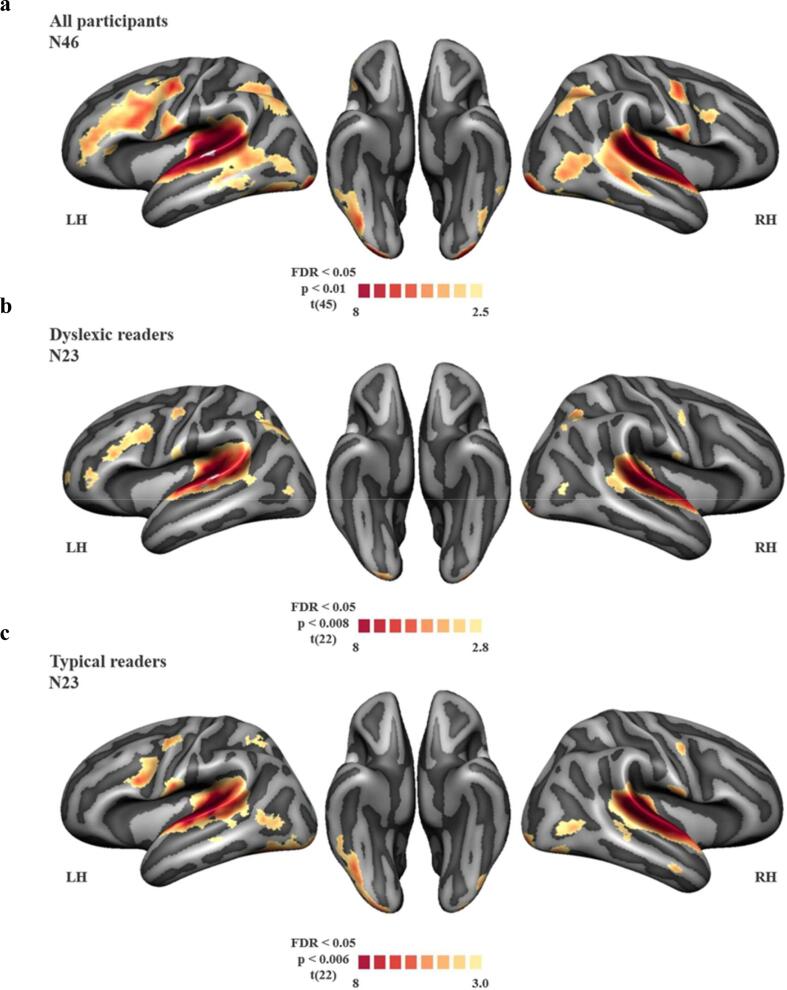
Cortical activation during the exposure blocks versus baseline in (a) all participants; (b) dyslexic readers; (c) typical readers.

During the exposure blocks, paired text and ambiguous speech sound stimuli evoked significant blood-oxygen-level-dependent (BOLD) responses in a broad bilateral network of brain areas typically associated with reading and audio-visual integration (Bonte et al., 2017, Dehaene et al., 2015, Shaywitz and Shaywitz, 2008, Van Atteveldt et al., 2004). These regions included the occipital cortex, (left) fusiform, bilateral superior temporal gyrus (STG), frontal and parietal areas (Fig. 4a). The Talairach coordinates of these activation clusters are reported in Table 2. The activation pattern was largely comparable between dyslexic and typical readers (Fig. 4b and c). Whole-brain comparisons of group differences between children with and without dyslexia, did not yield statistically significant results at the FDR < 0.05 level. We did, however observe significantly higher activation in the typical readers in a left hemisphere fusiform region at a more lenient voxel-level threshold of p < 0.01, corrected for multiple comparisons using a cluster threshold p < 0.05 that overlapped with the fusiform ROI.

**Table 2:** Talairach coordinates of cortical activation clusters during the audio-visual exposure blocks compared to baseline across groups.

Area	Hemisphere	Cluster size (n vertices)	Talairach coordinates (center of gravity)		
			x	y	z
Frontal	Left	1205	−49	16	37
STG/STS	Left	3580	−66	−34	14
Parietal	Left	1373	−45	−70	49
Lateral sensorimotor	Left	478	−67	−9	24
vOTC	Left	383	−51	−70	−13
V1	Left	142	−35	−103	−9
Frontal	Right	89	51	11	39
STG/STS	Right	2992	68	−26	14
Posterior MTG	Right	261	54	−74	7
Parietal	Right	769	46	−74	51
Lateral sensorimotor	Right	333	67	−8	24
vOTC	Right	86	47	−74	−13
V1	Right	181	35	−102	−6

STG = Superior Temporal Gyrus; STS = Superior Temporal Sulcus; vOTC = ventral Occipito-Temporal Cortex; V1 = primary visual cortex.

### 3.4 ROI-based group comparisons and correlations

**Fig. 5:**
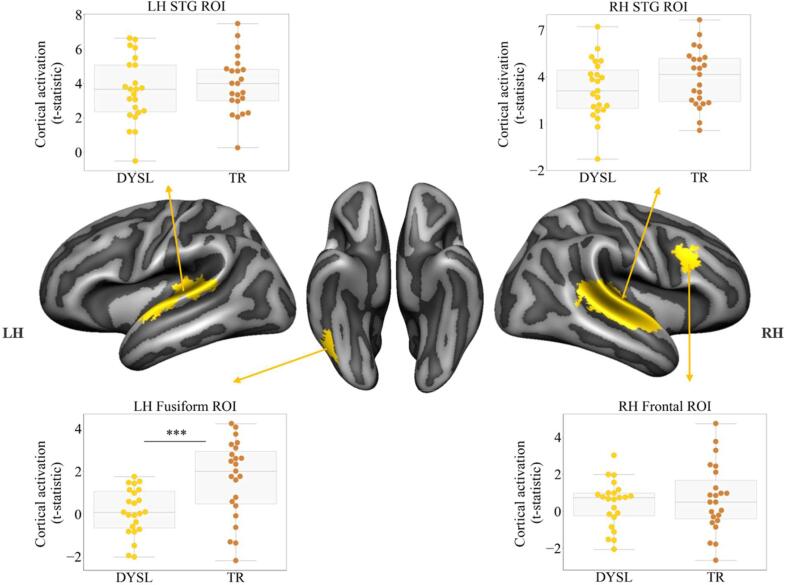
Group differences in cortical activation during the audio-visual exposure blocks within the regions of interest between dyslexic (DYSL; gold) and typical readers (TR; brown). Cortical activation is represented as individual t-statistics per participant (gold and brown dots) and group box-plots (grey) for each group. *** = *p* < 0.001.

To investigate group differences in brain regions typically associated with audio-visual processing of text and speech sounds, we performed additional ANOVA analyses within the bilateral STG, left fusiform and right frontal ROIs based on independently acquired data of the congruent condition in the passive listening/viewing task. The ROI comparisons yielded a significant activation difference in the left fusiform ROI [F(1,45) = 13.60, *p* < 0.01] with reduced activation in the dyslexic compared to typical readers. Cortical activation in the other ROIs did not differ between groups (Fig. 5). Additional ANCOVA analyses in all ROIs and for all three potential confounding variables (verbal and non-verbal WISC-III-NL sub-tests and age) yielded the same results, confirming that these variables did not significantly contribute to the observed (lack of) group differences.

To investigate whether our results were modulated by task performance – i.e. whether or not children show a recalibration effect – we performed the same group comparisons between dyslexic and typical readers in sub-groups of children who did show a text-based recalibration effect in the MRI (responders; 22 in total, 11 per group) and those children who did not (non-responders 24 in total, 12 per group). The analyses in responders replicated those observed in the full sample (see supplementary Fig. S2), showing that readers with dyslexia activate the left fusiform region less compared to typical readers even when they successfully recalibrated ambiguous speech perception towards the text stimuli. The analyses in non-responders did not show a group difference in the left fusiform region but did replicate the rest of our findings (see supplementary Fig. S3).

**Fig. 6:**
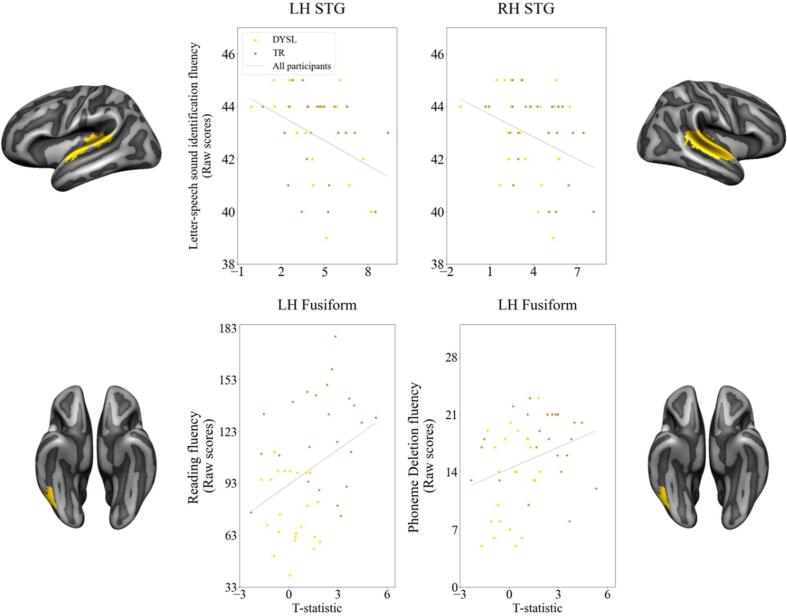
Results of the correlation analyses between cortical activation during the audio-visual exposure blocks within the regions of interest and children’s non-standardized reading scores; Top panel: bilateral STG; bottom panel: left fusiform region; DYSL = dyslexic readers; TR = typical readers.

We subsequently performed correlation analyses between activation within each of the four ROIs and children’s raw, non-standardised scores of letter-speech sound processing, reading and phonological skills. This yielded bilateral negative correlations between STG activation and letter-speech sound identification fluency (Fig. 6 top panel; left STG r(42) = −0.344, *p* < 0.05, q = 0.02; right STG r(42) = −0.300, *p* < 0.05, q = 0.02) as well as positive correlations between reading fluency (r(44) = 0.376, *p* < 0.01, q = 0.01) and phoneme deletion scores (r(44) = 0.307, *p* < 0.05, q = 0.02) and activation within the left fusiform ROI (Fig. 6 bottom panel).

## 4 Discussion

The present MRI study investigated text-based recalibration in 46 8–10 year-old children, half of whom had received an official diagnosis of dyslexia. Our fMRI findings showed activation within comparable brain areas in both groups during audio-visual exposure to letters and ambiguous speech sounds and comparable behavioural effects of text-based recalibration. A more detailed comparison did show significantly reduced activation within a left fusiform ROI for dyslexic compared to typical readers, which was correlated with children’s reading and phonological skills. Additionally, increased cortical activation in bilateral STG during exposure to text and ambiguous speech was linked to less fluent letter-speech sound identification, likely pointing to altered processing of the audio-visual stimuli in children with less automatized letter-speech sound associations.

Our behavioural results outside of the scanner corroborate previously reported behavioural findings showing significant recalibration effects regardless of dyslexia diagnosis (Romanovska et al., 2019). We did, however, observe slight differences in response proportions between the groups, with dyslexic readers being more likely to perceive the ambiguous post-test sounds as /ada/ compared to typical readers. Across both groups, the magnitude of the effect was reduced in the MRI scanner. This is likely due to contextual factors including scanner noise, sound quality in the MR-compatible headphones and unusual body position (performing the task lying down). Previous research has shown that the MRI environment reduces attentional focus on the task (van Maanen et al., 2016) and 8–10 year old children who are still developing their attentional skills (Amso and Scerif, 2015, Betts et al., 2006, Klenberg et al., 2001, Klimkeit et al., 2004, Lin et al., 1999) may be more prone to such effects. Although somewhat reduced compared to offline behavioural experiments, in adults behavioural recalibration effects tend to be preserved in the MRI setting (Bonte et al., 2017, Kilian-Hütten et al., 2011a, Ullas et al., 2020b). Thus, a developmental trajectory of both the text-based recalibration effect and more general cognitive and attentional mechanisms may underlie the differences in text-based recalibration performance in the MRI environment between children and adults. Future research in larger groups of adults and/or older children who more consistently show a significant text-based recalibration effect in the MRI should aim to elucidate the associations between the magnitude of the recalibration effect and cortical activation. An inspection of individual behavioural performance in the scanner revealed that about half of the children in each group did show a recalibration effect. Intriguingly, unlike previous behavioural results in adults with dyslexia (Keetels et al., 2018), there was no relation between children’s (non)responsiveness to recalibration and reading skills.

In terms of cortical activation, a broad bilateral network of brain areas typically associated with reading and audio-visual integration was seen, including bilateral STG, frontal and parietal brain areas. These regions overlap with those reported in a previous fMRI study employing text-based recalibration in adults (Bonte et al., 2017), as well as in studies investigating cortical responses to letters and speech sounds in children and adults (Blau et al., 2010, Blau et al., 2009, Chyl et al., 2017, Kronschnabel et al., 2014, McNorgan et al., 2014, McNorgan and Booth, 2015, Plewko et al., 2018). Moreover, our results suggest that dyslexic and typical readers recruit a comparable network of cortical areas during audio-visual exposure to text and ambiguous speech sounds. The observed similarities in the brain areas activated in our study and the recalibration study in adults indicates that this network is already in place in 8–10 year-old children.

Despite this similarity at the whole brain level, our subsequent ROI analyses showed a significant reduction in brain activation in dyslexic compared to typically reading children in a region that is involved in the visual processing of text – the left fusiform (Dehaene and Cohen, 2011, Dehaene and Dehaene-Lambertz, 2016, Dehaene et al., 2010, Dehaene-Lambertz et al., 2018, Monzalvo and Dehaene-Lambertz, 2013). The observed group difference remained significant in an additional analysis in a subgroup of children (N = 22, 11 dyslexic readers) who did show a text-based recalibration effect in the MRI scanner. This finding is in line with previous studies reporting under-activation of the left ventral occipito-temporal cortex in readers with dyslexia (Dehaene and Cohen, 2011, Hoeft et al., 2007, Paulesu, 2001, Richlan et al., 2009, Wimmer et al., 2010), as well as at-risk pre-readers (Centanni et al., 2019, Karipidis et al., 2017, Plewko et al., 2018). Activation within this ROI was furthermore positively associated with reading fluency and phoneme deletion, indicating that better reading and phonological skills were linked to increased left fusiform activation during audio-visual exposure to letters and ambiguous speech sounds. This finding corroborates and extends previous research reporting an association between reading fluency and accuracy and cortical activation in this region in response to text (Ben-Shachar et al., 2011, Blau et al., 2010).

The positive association between activation in the left fusiform ROI with phoneme deletion and reading scores likely reflects the ongoing refinement of letter-speech sound coupling in children within our age range. Areas in the left fusiform gyrus have been found to play a role in text-speech coupling (Graves et al., 2010), categorical perception of phonemes (Conant et al., 2014), and to be modulated by auditory stimuli (McNorgan and Booth, 2015). This may be even more so in children, as previous developmental studies report more overlap in activation for visual and auditory tasks in unimodal brain areas in children compared to adults (Booth et al., 2001) as well as a transformation of bilateral higher order visual areas from multimodal to unimodal processing over the course of (reading) development (Church et al., 2008). Thus, we may conclude that the observed group difference in cortical activation between dyslexic and typical readers in this ROI was driven by children’s reading and phonological skills and adds to the body of research showing altered processing of letters, and their mapping to speech sounds, in the left fusiform in children with dyslexia.

The comparable behavioural performance on the text-based recalibration task in children with and without dyslexia despite differences in brain activation remains to be explained. A possible interpretation could be that children with dyslexia rely more on a dorsal, more explicit reading cortical system involved in mapping letters and speech sounds and have not yet made the switch to the more automatized ventral cortical system involving the left fusiform (Pugh et al., 2001, Sandak et al., 2004). Indeed, a longitudinal study in children with and without dyslexia reported a later refinement of the ventral occipito-temporal cortex in the dyslexic readers (Morken et al., 2017). Moreover, cross-sectional studies investigating connectivity between the ventral and dorsal reading systems report aberrant connectivity in dyslexic compared to typical readers (Finn et al., 2014, Schurz et al., 2015, van der Mark et al., 2011). Although this hypothesis will need to be investigated in future studies, the dyslexic children may have achieved similar task performance through a subtle difference in the involvement of ventral versus dorsal cortical networks compared to their typically reading peers. This pattern of different neural recruitment despite similar behavioral performance is further supported by the presence of reduced left fusiform activation in dyslexic versus typical readers when restricting the analysis to those children who did show text-based recalibration in the scanner.

A potential explanation for the observed group differences in left fusiform activation despite comparable task performance could be that functional and structural connectivity between the vOTC and dorsal brain regions involved in speech processing develop differently in children with reading difficulties, likely as a result of a variety of risk- and protective factors (Ozernov-Palchik and Gaab, 2016, Perry et al., 2019, Zuk et al., 2020). It has been proposed that structural connectivity patterns between the text-sensitive visual word form area (VWFA) in the vOTC and the dorsal reading (speech processing) system are established prior to formal reading instruction in pre-reading children around the age of 5 (Saygin et al., 2016). A study in children of the same age at familial risk for developing dyslexia has furthermore shown that at-risk children who go on to develop reading difficulties, show less activation in this region compared to both at-risk children who become typical readers and children without a familial risk (Centanni et al., 2019). Furthermore, developmental changes in functional connectivity patterns between VWFA and the dorsal reading system have been shown to parallel gains in reading fluency (Wise-Younger et al., 2017). Thus, if the connectivity patterns are already established at the pre-reader stage and those children who go on to struggle with reading show less activation in the left vOTC early on, aberrant connectivity patterns between the ventral and dorsal reading systems may have contributed to and/or underlie differences in functional activity during letter-speech sound processing as observed in the current study.

Unlike previous studies (Blau et al., 2010, Blau et al., 2009, Monzalvo et al., 2012), we did not find significantly reduced superior temporal cortical activity in dyslexic versus typically reading children, and this activity also did not scale with individual differences in reading and/or phonological skills (Bonte et al., 2016, Brennan et al., 2013, Conant et al., 2014). This discrepancy could relate to the type of task employed (i.e. recalibration task instead of letter-speech sound congruency manipulation), use of ambiguous speech stimuli, characteristics of our dyslexia sample (children at the beginning of remediation focused on letter-speech sound automatization), or family history of dyslexia (Hakvoort et al., 2014, Vandermosten et al., 2020). Interestingly, our findings showed the opposite pattern where less fluent letter-speech sound identification was related to increased bilateral STC activation. This implies stronger involvement of the bilateral STC during the processing of letters and (ambiguous) speech sounds in children who are slower in the audio-visual mapping of these type of stimuli. This stronger involvement of the bilateral STC could be a result of the principle of inverse effectiveness (Wallace et al., 1996). This principle postulates that multi-sensory integration is the highest when stimuli from the two modalities are weak. The inverse effectiveness principle has been observed in the STS as an increased response to degraded audio-visual lip-read words (Stevenson and James, 2009) and in degraded audio-visual sentence comprehension behaviourally (van de Rijt et al., 2019). While the visual stimuli in our study are clear, the auditory stimuli are ambiguous and may thus be considered “weaker” in terms of sensory input. The visual text could therefore be used to facilitate the auditory stimulus processing, increasing neural interaction and improving the stimulus identity prediction (Van Atteveldt et al., 2014). However, this facilitation might not be as profound in children with less fluent (i.e. automatic) letter-speech sound mapping. Thus, we might speculate that children with lower scores on the letter-speech sound identification task may have benefited more from inputs to both, the auditory and visual modality during letter-speech sound integration, resulting in the observed increase in bilateral auditory cortical activation. Another factor that influences multi-sensory integration is cue reliability, i.e. which cue is relevant for a given task (Van Atteveldt et al., 2014). Children with more automatized letter-speech sound representations may be better equipped to weigh the clear visual stimulus (“aba”/ “ada” text) as the most reliable one, therefore facilitating audio-visual integration, possibly resulting in less cortical activation. Thus, our findings imply that (1) left-fusiform activation during audio-visual exposure to letters and (ambiguous) speech sounds scales with inter-individual differences in children’s reading and phonological skills, and (2) increased bilateral STG activation may be required for (comparable) audio-visual integration in children with less automatic letter-speech sound representations.

## 5 Conclusion

The current fMRI study investigated text-based recalibration in 8–10 year-old children with and without dyslexia. Our results revealed that children within this age-group show a significant recalibration effect regardless of dyslexia diagnosis. Nevertheless, group comparisons within key reading and audio-visual integration ROIs revealed significantly higher activation in a left fusiform ROI in typical readers compared to children with dyslexia, which correlated with children’s reading and phonological skills. These findings corroborate previous research indicating altered functionality of text-sensitive left occipito-temporal cortex in dyslexic readers. The correlation analyses also showed differences in brain activation patterns in bilateral STG with more activation seen in children with poorer performance on a letter-speech sound identification fluency task. While speculative, we believe that this negative association may be linked to differential processing of the audio-visual information in children with less automatized letter-speech sound mapping. Subsequent investigations of changes in cortical activation and behavioural performance within the same cohort longitudinally will enable exploration of inter-individual differences within and across groups as their reading skills develop.

## CRediT authorship contribution statement

**Linda Romanovska:** Conceptualization, Software, Formal analysis, Investigation, Resources, Writing - original draft, Writing - review & editing, Visualization, Project administration. **Roef Janssen:** Formal analysis, Investigation, Resources, Project administration. **Milene Bonte:** Conceptualization, Resources, Writing - original draft, Writing - review & editing, Supervision, Funding acquisition.

## Declaration of Competing Interest

The authors declare that they have no known competing financial interests or personal relationships that could have appeared to influence the work reported in this paper.

## References

[b0005] Amso D., Scerif G. (2015). The attentive brain: Insights from developmental cognitive neuroscience. Nat. Rev. Neurosci..

[b0010] Aravena S. (2017). Letter-Speech Sound Learning in Children with Dyslexia From Behavioral Research to Clinical Practice.

[b0015] Astrom R.L., Wadsworth S.J., DeFries J.C. (2007). Etiology of the stability of reading difficulties: the longitudinal twin study of reading disabilities. Twin Res. Human Genetics.

[b0020] Ben-Shachar M., Dougherty R.F., Deutsch G.K., Wandell B.A. (2011). The development of cortical sensitivity to visual word forms. J. Cognit. Neurosci..

[b0025] Bertelson P., Vroomen J., De Gelder B. (2003). Visual recalibration of auditory speech identification: a McGurk aftereffect. Psychol. Sci..

[b0030] Betts J., Mckay J., Maruff P., Anderson V. (2006). The development of sustained attention in children: the effect of age and task load. Child Neuropsychol..

[b0035] Blau V., Reithler J., Van Atteveldt N., Seitz J., Gerretsen P., Goebel R., Blomert L. (2010). Deviant processing of letters and speech sounds as proximate cause of reading failure: a functional magnetic resonance imaging study of dyslexic children. Brain.

[b0040] Blau V., van Atteveldt N., Ekkebus M., Goebel R., Blomert L. (2009). Reduced neural integration of letters and speech sounds links phonological and reading deficits in adult dyslexia. Curr. Biol..

[b0045] Blomert L. (2011). The neural signature of orthographic-phonological binding in successful and failing reading development. NeuroImage.

[b0050] Blomert L., Vaessen A. (2009). 3DM Differential Diagnostics for Dyslexia: Cognitive Analysis of Reading and Spelling.

[b0055] Blomert L., Willems G. (2010). Is there a causal link from a phonological awareness deficit to reading failure in children at familial risk for dyslexia?. Dyslexia.

[b0060] Boersma P., Weenink D.J.M. (2001). PRAAT: doing phonetics by computer. Glot Int..

[b0065] Bonte M., Correia J., Keetels M., Vroomen J., Formisano E. (2017). Reading-induced shifts of perceptual speech representations in auditory cortex. Sci. Rep..

[b0070] Bonte M.L., Poelmans H., Blomert L. (2007). Deviant neurophysiological responses to phonological regularities in speech in dyslexic children. Neuropsychologia.

[b0075] Bonte M., Hausfeld L., Scharke W., Valente G., Formisano E. (2014). Task-dependent decoding of speaker and vowel identity from auditory cortical response patterns. J. Neurosci..

[b0080] Bonte M., Ley A., Scharke W., Formisano E. (2016). Developmental refinement of cortical systems for speech and voice processing. NeuroImage.

[b0085] Booth J.R., Burman D.D., Van Santen F.W., Harasaki Y., Gitelman D.R., Parrish T.B., Mesulam M.M. (2001). The development of specialized brain systems in reading and oral-language. Child Neuropsychol..

[b0090] Brem S., Halder P., Bucher K., Summers P., Martin E., Brandeis D. (2009). Tuning of the visual word processing system: distinct developmental ERP and fMRI effects. Hum. Brain Mapp..

[b0095] Brennan C., Cao F., Pedroarena-Leal N., McNorgan C., Booth J.R. (2013). Reading acquisition reorganizes the phonological awareness network only in alphabetic writing systems. Hum. Brain Mapp..

[b0100] Centanni T.M., Norton E.S., Ozernov-Palchik O., Park A., Beach S.D., Halverson K., Gabrieli J.D.E. (2019). Disrupted left fusiform response to print in beginning kindergartners is associated with subsequent reading. NeuroImage: Clinical.

[b0105] Church J.A., Coalson R.S., Lugar H.M., Petersen S.E., Schlaggar B.L. (2008). A developmental fMRI study of reading and repetition reveals changes in phonological and visual mechanisms over age. Cereb. Cortex.

[b0110] Chyl K., Kossowski B., Dębska A., Łuniewska M., Banaszkiewicz A., Żelechowska A., Jednoróg K. (2017). Prereader to beginning reader: changes induced by reading acquisition in print and speech brain networks. J. Child Psychol. Psychiatry.

[b0115] Clayton F.J., Hulme C. (2017). Automatic activation of sounds by letters occurs early in development but is not impaired in children with dyslexia. Sci. Stud. Read..

[b0120] Conant L.L., Liebenthal E., Desai A., Binder J.R. (2014). FMRI of phonemic perception and its relationship to reading development in elementary- to middle-school-age children. NeuroImage.

[b0125] Correia J.M., Jansma B.M.B., Bonte M. (2015). Decoding articulatory features from fMRI responses in dorsal speech regions. J. Neurosci..

[b0130] Dehaene-Lambertz G., Monzalvo K., Dehaene S. (2018). The emergence of the visual word form: longitudinal evolution of category-specific ventral visual areas during reading acquisition. PLoS Biol..

[b0135] Dehaene S., Cohen L. (2011). The unique role of the visual word form area in reading. Trends Cognitive Sci..

[b0140] Dehaene S., Cohen L., Morais J., Kolinsky R. (2015). Illiterate to literate: behavioural and cerebral changes induced by reading acquisition. Nat. Rev. Neurosci..

[b0145] Dehaene S., Dehaene-Lambertz G. (2016). Is the brain prewired for letters?. Nat. Neurosci..

[b0150] Dehaene S., Pegado F., Braga L.W., Ventura P., Nunes Filho G., Jobert A., Cohen L. (2010). How learning to read changes the cortical networks for vision and language. Science.

[b0155] Ehri L.C., Snowling M.J., Hulme C. (2005). Development of sight word reading: phases and findings. The Science of Reading: A Handbook.

[b0160] Finn E.S., Shen X., Holahan J.M., Scheinost D., Lacadie C., Papademetris X., Constable R.T. (2014). Disruption of functional networks in dyslexia: a whole-brain, data-driven analysis of connectivity. Biol. Psychiatry.

[b0165] Fraga González, G., 2015. Fixing fluency: Neurocognitive assessment of a dysfluent reading intervention. Ph.D. thesis, Psychology Research Institute, Amsterdam. https://doi.org/10.1177/1745691612459060.

[b0170] Frost M.A., Goebel R. (2012). Measuring structural-functional correspondence: Spatial variability of specialised brain regions after macro-anatomical alignment. NeuroImage.

[b0175] Froyen D., Van Atteveldt N., Bonte M., Blomert L. (2008). Cross-modal enhancement of the MMN to speech-sounds indicates early and automatic integration of letters and speech-sounds. Neurosci. Lett..

[b0180] Goswami U. (2003). Why theories about developmental dyslexia require developmental designs. Trends Cognitive Sci..

[b0185] Graves W.W., Desai R., Humphries C., Seidenberg M.S., Binder J.R. (2010). Neural systems for reading aloud: a multiparametric approach. Cereb. Cortex.

[b0190] Hakvoort B., van der Leij A., Maurits N., Maassen B., van Zuijen T.L. (2014). Basic auditory processing is related to familial risk, not to reading fluency: An ERP study. Cortex.

[b0195] Hoeft F., Meyler A., Hernandez A., Juel C., Taylor-Hill H., Martindale J.L., Gabrieli J.D.E. (2007). Functional and morphometric brain dissociation between dyslexia and reading ability. PNAS.

[b0200] Karipidis I., Pleisch G., Röthlisberger M., Hofstetter C., Dornbierer D., Stämpfli P., Brem S. (2017). Neural initialization of audiovisual integration in prereaders at varying risk for developmental dyslexia. Hum. Brain Mapp..

[b0205] Keetels M., Bonte M., Vroomen J. (2018). A selective deficit in phonetic recalibration by text in developmental dyslexia. Front. Psychol..

[b0210] Keetels M., Schakel L., Bonte M., Vroomen J. (2016). Phonetic recalibration of speech by text. Attention, Percept. Psychophys..

[b0215] Kilian-Hütten N., Valente G., Vroomen J., Formisano E. (2011). Auditory cortex encodes the perceptual interpretation of ambiguous sound. J. Neurosci..

[b0220] Kilian-Hütten N., Vroomen J., Formisano E. (2011). Brain activation during audiovisual exposure anticipates future perception of ambiguous speech. NeuroImage.

[b0225] Klenberg L., Korkman M., Lahti-Nuuttila P. (2001). Differential development of attention and executive functions in 3- to 12-year-old Finnish children. Developm. Neuropsychol..

[b0230] Klimkeit E.I., Mattingley J.B., Sheppard D.M., Farrow M., Bradshaw J.L. (2004). Examining the development of attention and executive functions in children with a novel paradigm. Child Neuropsychol..

[b0235] Kort, W., Schittekatte, M., Bosmans, M., Compaan, E. L., Dekker, P. H., Vermeir, G., & Verhaeghe, P. (2005). WISC-III-NL. (W. Kort, M. Schittekatte, M. Bosmans, E. L. Compaan, P. H. Dekker, G. Vermeir, & P. Verhaeghe, Eds.) (Trans.). Harcourt Test Publishers, London.

[b0240] Kronschnabel J., Brem S., Maurer U., Brandeis D. (2014). The level of audiovisual print-speech integration deficits in dyslexia. Neuropsychologia.

[b0245] Lin C.C.H., Hsiao C.K. (1999). Development of sustained attention assessed using the continuous performance test among children 6–15 years of age. J. Abnorm. Child Psychol..

[b0250] Lyon G.R., Shaywitz S.E., Shaywitz B.A. (2003). A definition of dyslexia. Ann. Dyslexia.

[b0255] Maurer U., Brem S., Kranz F., Bucher K., Benz R., Halder P., Brandeis D. (2006). Coarse neural tuning for print peaks when children learn to read. NeuroImage.

[b0260] Maurer U., Zevin J.D., McCandliss B.D. (2008). Left-lateralized N170 effects of visual expertise in reading: evidence from japanese syllabic and logographic scripts. J. Cognit. Neurosci..

[b0265] McNorgan C., Awati N., Desroches A.S., Booth J.R. (2014). Multimodal lexical processing in auditory cortex is literacy skill dependent. Cereb. Cortex.

[b0270] McNorgan C., Booth J.R. (2015). Skill dependent audiovisual integration in the fusiform induces repetition suppression. Brain Lang..

[b0275] Monzalvo K., Dehaene-Lambertz G. (2013). How reading acquisition changes children’s spoken language network. Brain Lang..

[b0280] Monzalvo K., Fluss J., Billard C., Dehaene S., Dehaene-Lambertz G. (2012). Cortical networks for vision and language in dyslexic and normal children of variable socio-economic status. NeuroImage.

[b0285] Morken, F., Helland, T., Hugdahl, K., Specht, K., 2017. Reading in dyslexia across literacy development: A longitudinal study of effective connectivity. NeuroImage, 144(September 2016), 92–100. https://doi.org/10.1016/j.neuroimage.2016.09.060. 10.1016/j.neuroimage.2016.09.060 27688204

[b0290] Nash H.M., Gooch D., Hulme C., Mahajan Y., Mcarthur G., Steinmetzger K., Snowling M.J. (2016). Are the literacy difficulties that characterize developmental dyslexia associated with a failure to integrate letters and speech sounds?. Develop. Sci..

[b0295] Noordenbos M.W., Serniclaes W. (2015). The categorical perception deficit in dyslexia: a meta-analysis. Sci. Stud. Read..

[b0300] Norris D., McQueen J.M., Cutler A. (2003). Perceptual learning in speech. Cogn. Psychol..

[b0305] Ozernov-Palchik O., Gaab N. (2016). Tackling the “dyslexia paradox”: Reading brain and behavior for early markers of developmental dyslexia. Wiley Interdiscip. Rev. Cognit. Sci..

[b0310] Paulesu E. (2001). Dyslexia: cultural diversity and biological unity. Science.

[b0315] Pennington B.F. (2006). From single to multiple deficit models of developmental disorders. Cognition.

[b0320] Perry C., Zorzi M., Ziegler J.C. (2019). Understanding dyslexia through personalized large-scale computational models. Psychol. Sci..

[b0325] Peterson R.L., Pennington B.F. (2012). Seminar: developmental dyslexia. Lancet.

[b0330] Plewko J., Chyl K., Bola Ł., Łuniewska M., Dębska A., Banaszkiewicz A., Jednoróg K. (2018). Letter and speech sound association in emerging readers with familial risk of dyslexia. Front. Hum. Neurosci..

[b0335] Price C.J., Devlin J.T. (2011). The interactive account of ventral occipitotemporal contributions to reading. Trends Cognitive Sci..

[b0340] Pugh K.R., Mencl W.E., Jenner A.R., Katz L., Frost S.J., Lee J.R., Shaywitz B.A. (2001). Neurobiological studies of reading and reading disability. J. Commun. Disord..

[b0345] Ramus F., Szenkovits G. (2008). What phonological deficit?. Q. J. Exp. Psychol..

[b0350] Richlan F., Kronbichler M., Wimmer H. (2009). Functional abnormalities in the dyslexic brain: a quantitative meta-analysis of neuroimaging studies. Hum. Brain Mapp..

[b0355] Romanovska L., Janssen R., Bonte M. (2019). Reading-induced shifts in speech perception in dyslexic and typically reading children. Front. Psychol..

[b0360] Samuel A.G., Kraljic T. (2009). Perceptual learning for speech. Attention, Percept. Psychophys..

[b0365] Sandak R., Mencl E.E., Frost S.J., Pugh K.R. (2004). The neurobiological basis of skilled and impaired reading: recent findings and new directions. Sci. Stud. Read..

[b0370] Schlaggar B.L., McCandliss B.D. (2007). Development of neural systems for reading. Annu. Rev. Neurosci..

[b0375] Schumacher J., Hoffmann P., Schmäl C., Schulte-Körne G., Nöthen M.M. (2007). Genetics of dyslexia: the evolving landscape. J. Med. Genet..

[b0380] Schurz M., Wimmer H., Richlan F., Ludersdorfer P., Klackl J., Kronbichler M. (2015). Resting-state and task-based functional brain connectivity in developmental dyslexia. Cereb. Cortex.

[b0385] Scott M. (2016). Speech imagery recalibrates speech-perception boundaries. Attention, Percept. Psychophys..

[b0390] Shaywitz S.E., Shaywitz B.A. (2008). Paying attention to reading: the neurobiology of reading and dyslexia. Dev. Psychopathol..

[b0395] Shaywitz S.E., Shaywitz B.A., Pugh K.R., Fulbright R.K., Constable R.T., Mencl W.E., Gore J.C. (1998). Functional disruption in the organization of the brain for reading in dyslexia. PNAS.

[b0400] Snowling M.J. (1980). The development of grapheme-phoneme correspondence in normal and dyslexic readers. J. Exp. Child Psychol..

[b0405] Snowling M.J. (2013). Early identification and interventions for dyslexia: a contemporary view. J. Res. Special Educ. Needs.

[b0410] Snowling M.J., Melby-Lervåg M. (2016). Oral language deficits in familial dyslexia: a meta-analysis and review. Psychol. Bull..

[b0415] Stevenson R.A., James T.W. (2009). Audiovisual integration in human superior temporal sulcus: inverse effectiveness and the neural processing of speech and object recognition. NeuroImage.

[b0420] Talairach, & Tournoux. (1988). Co-Planar Stereotaxic Atlas of the Human Brain.

[b0425] Ullas S., Formisano E., Eisner F., Cutler A. (2020). Interleaved lexical and audiovisual information can retune phoneme boundaries. Attention, Percept. Psychophys..

[b0430] Ullas S., Hausfeld L., Cutler A., Eisner F., Formisano E. (2020). Neural correlates of phonetic adaptation as induced by lexical and audiovisual context. J. Cognit. Neurosci..

[b0435] Van Atteveldt N., Ansari D. (2014). How symbols transform brain function: a review in memory of Leo Blomert. Trends Neurosci. Educ..

[b0440] Van Atteveldt N., Formisano E., Goebel R., Blomert L. (2004). Integration of letters and speech sounds in the human brain. Neuron.

[b0445] Van Atteveldt N., Murray M.M., Thut G., Schroeder C.E. (2014). Multisensory integration: Flexible use of general operations. Neuron.

[b0450] Van Bergen E., De Jong P.F., Plakas A., Maassen B., Van Der Leij A. (2012). Child and parental literacy levels within families with a history of dyslexia. J. Child Psychol. Psychiatry.

[b0455] van Bergen E., van der Leij A., de Jong P.F. (2014). The intergenerational multiple deficit model and the case of dyslexia. Front. Hum. Neurosci..

[b0460] van de Rijt L.P.H., Roye A., Mylanus E.A.M., van Opstal A.J., van Wanrooij M.M. (2019). The principle of inverse effectiveness in audiovisual speech perception. Front. Hum. Neurosci..

[b0465] van der Mark S., Klaver P., Bucher K., Maurer U., Schulz E., Brem S., Brandeis D. (2011). The left occipitotemporal system in reading: disruption of focal fMRI connectivity to left inferior frontal and inferior parietal language areas in children with dyslexia. NeuroImage.

[b0470] van Linden S., Vroomen J. (2008). Audiovisual speech recalibration in children. J. Child Language.

[b0475] van Maanen L., Forstmann B.U., Keuken M.C., Wagenmakers E.J., Heathcote A. (2016). The impact of MRI scanner environment on perceptual decision-making. Behavior Res. Methods.

[b0480] Vandermosten M., Correia J., Vanderauwera J., Wouters J., Ghesquière P., Bonte M. (2020). Brain activity patterns of phonemic representations are atypical in beginning readers with family risk for dyslexia. Develop. Sci..

[b0485] Vroomen J., Baart, Micah M.T.W., Murray M. (2012). Phonetic recalibration in audiovisual speech. The Neural Bases of Multisensory Processes.

[b0490] Vroomen J., Van Linden S., Keetels M., De Gelder B., Bertelson P. (2004). Selective adaptation and recalibration of auditory speech by lipread information: Dissipation. Speech Commun..

[b0495] Wallace M.T., Wilkinson L.K., Stein B.E. (1996). Representation and integration of multiple sensory inputs in primate superior colliculus. J. Neurophysiol..

[b0500] Wimmer H., Schurz M., Sturm D., Richlan F., Klackl J., Kronbichler M., Ladurner G. (2010). A dual-route perspective on poor reading in a regular orthography: an fMRI study. Cortex.

[b0505] Ye Z., Rüsseler J., Gerth I., Münte T.F. (2017). Audiovisual speech integration in the superior temporal region is dysfunctional in dyslexia. Neuroscience.

[b0510] Žarić G., González G.F., Tijms J., Van Der Molen M.W., Blomert L., Bonte M. (2014). Reduced neural integration of letters and speech sounds in dyslexic children scales with individual differences in reading fluency. PLoS ONE.

[b0515] Zuk, J., Dunstan, J., Norton, E., Yu, X., Ozernov-Palchik, O., Wang, Y., Gaab, N., 2020. Multifactorial pathways facilitate resilience among kindergarteners at risk for dyslexia: A longitudinal behavioral and neuroimaging study. Developmental Science, (April 2019), 1–18. https://doi.org/10.1111/desc.12983. 10.1111/desc.12983 PMC7606625 32356911

