# Supplementary material for: Cortical responses to letters and ambiguous speech vary with reading skills in dyslexic and typically reading children
Source: Neuroimage Clin. 2021 Feb 12;30:102588. doi: 10.1016/j.nicl.2021.102588 (PMC7907898; doi:10.1016/j.nicl.2021.102588)
Supplement: Supplementary data 1 [file mmc1.docx]

**Supplementary materials**

**
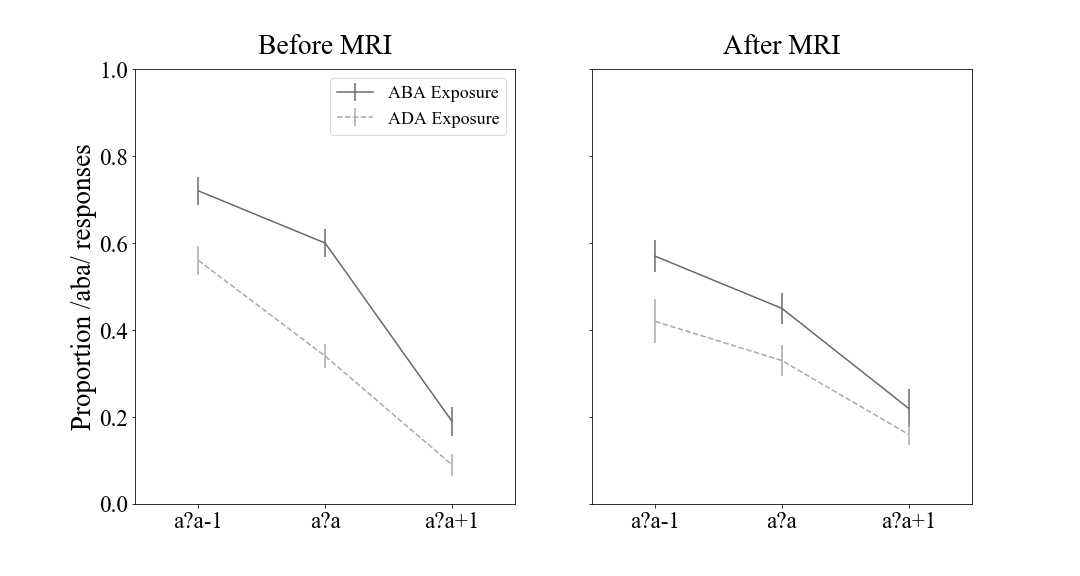
**

S1: /aba/ response proportions for the /a?a/-1, /a?a/ and /a?a/+1 post-test sounds following an “aba” versus “ada” exposure block in children who completed the behavioural experiment before the MRI session (left) and children who completed it afterwards (right).

**
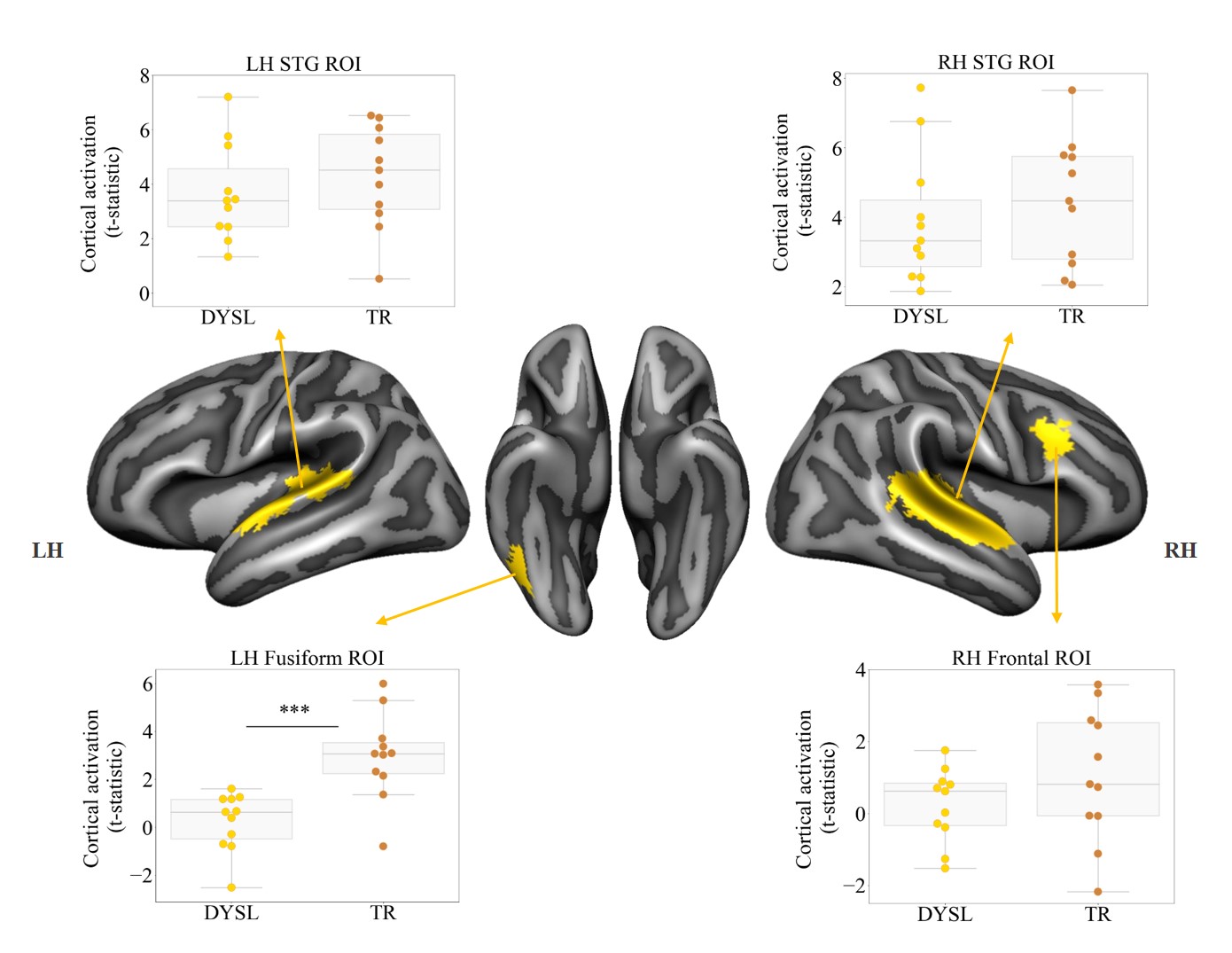
**

S2: Group differences in cortical activation during the audio-visual exposure blocks within the regions of interest between dyslexic (DYSL; gold) and typical readers (TR; brown) who show a text-based recalibration effect in the MRI scanner. Cortical activation is represented as individual t-statistics per participant (gold and brown dots) and group box-plots (grey) for each group. *** = *p* < 0.001


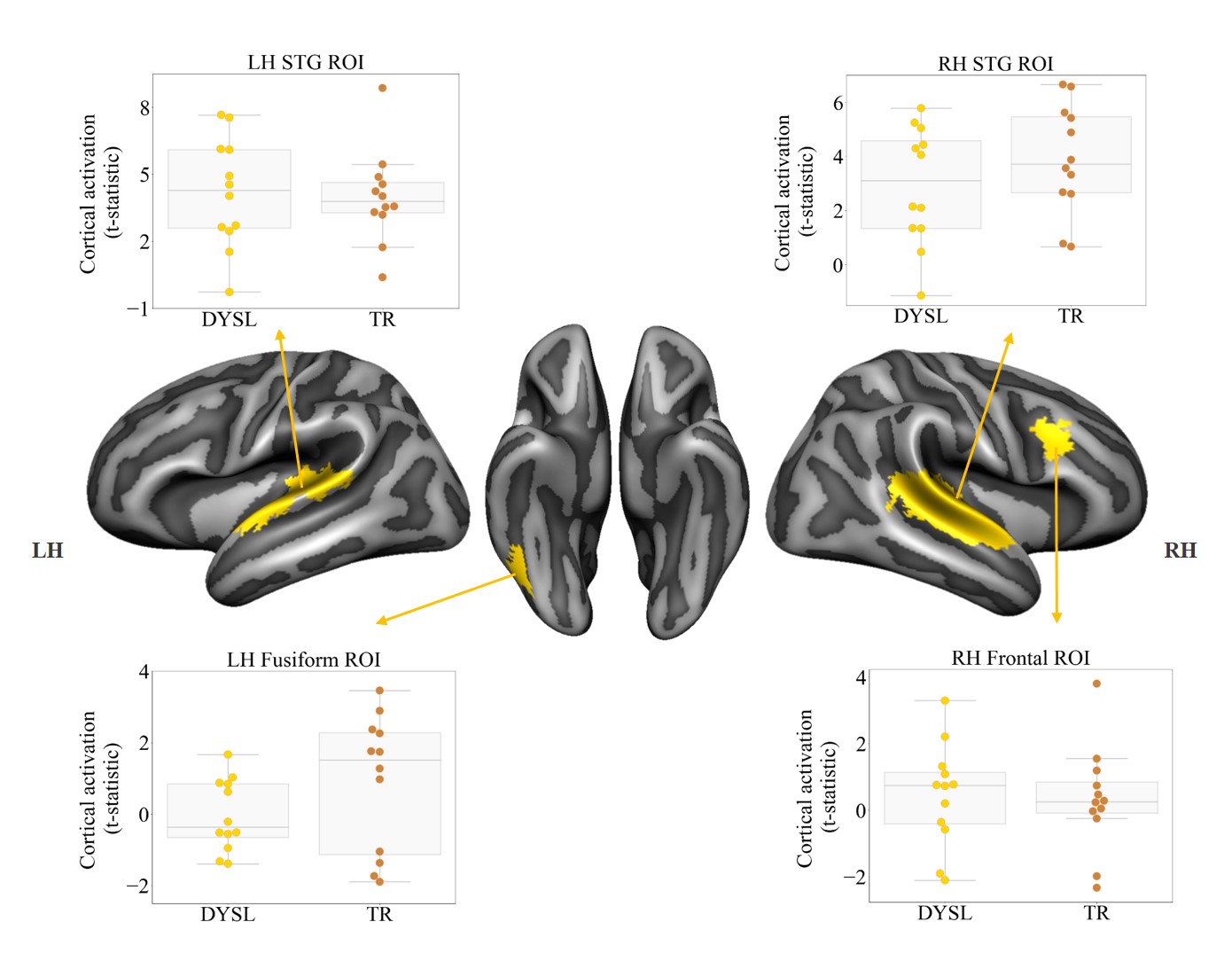


S3: Group differences in cortical activation during the audio-visual exposure blocks within the regions of interest between dyslexic (DYSL; gold) and typical readers (TR; brown) who do not show a text-based recalibration effect in the MRI scanner. Cortical activation is represented as individual t-statistics per participant (gold and brown dots) and group box-plots (grey) for each group.
